# Evaluating Oncological Outcomes in Patients with Multiple PiRADS Lesion Treated with Robot-Assisted Radical Prostatectomy for Prostate Cancer: Results from a Large Contemporary Cohort with Centralized MpMRI Evaluation in a High-Volume Center

**DOI:** 10.3390/jcm15103787

**Published:** 2026-05-14

**Authors:** Luca Lambertini, Simone Sforza, Filippo Lipparini, Marco Saladino, Fabrizio Di Maida, Antonio Andrea Grosso, Giulia Carli, Francesca Conte, Anna Cadenar, Beatrice Giustozzi, Francesco Lasorsa, Mattia Lo Re, Rino Oriti, Riccardo Fantechi, Gianni Vittori, Andrea Minervini, Andrea Mari

**Affiliations:** 1 Department of Experimental and Clinical Medicine, University of Florence, 50121 Firenze, Italy; simone.sforza@unifi.it (S.S.); filippo.lipparini@unifi.it (F.L.); marco.saladino@unifi.it (M.S.); fabrizio.dimaida@unifi.it (F.D.M.); antonioandrea.grosso@unifi.it (A.A.G.); carli.giulia96@gmail.com (G.C.); francesca.conte1@unifi.it (F.C.); anna.cadenar@unifi.it (A.C.); beatrice.giustozzi@unifi.it (B.G.); mattia.lore@unifi.it (M.L.R.); rino.oriti@unifi.it (R.O.); riccardo.fantechi@unifi.it (R.F.); gianni.vittori@unifi.it (G.V.); andrea.minervini@unifi.it (A.M.); andrea.mari@unifi.it (A.M.); 2 Unit of Oncologic, Minimally-Invasive Robotic Urology and Andrology, Careggi Hospital, 50134 Florence, Italy; 3 Urology, Andrology and Kidney Transplantation Unit, Department of Precision and Regenerative Medicine and Ionian Area, University of Bari “Aldo Moro”, 70121 Bari, Italy

**Keywords:** prostate cancer, magnetic resonance imaging, PIRADS

## Abstract

**Objective**: To evaluate the early oncological outcomes of patients treated with robot-assisted radical prostatectomy for prostate cancer with multiple PIRADS lesions at baseline mpMRI in a tertiary referral center. **Methods**: Data of consecutive patients undergoing robot-assisted radical prostatectomy between 2020 and 2022 at a high-volume tertiary referral center were prospectively collected. mpMRI data was evaluated by two expert uro-radiologists at our center. All patients received an MRI–ultrasound fusion biopsy. **Results**: Overall, 286 patients with multiple PIRADS lesions treated with robot-assisted radical prostatectomy at a tertiary referral center were included. Unilateral and bilateral nerve-sparing were achieved in 63 (22.3%) and 124 (43.1%) patients, respectively. Median age was 69 years (IQR: 64–72), while median Charlson Comorbidity Index was 3 (IQR: 2–4). The presence of two PIRADS lesions was found in the 81.8% of cases, while 18.2% presented with three or more. Bilateral lesions were observed in 67.4% of cases. The dominant lesion was PIRADS 4 in 57.3% and PIRADS 5 in 32.3% of cases, with a median diameter of 12 mm (IQR: 10–17). Pathological upstaging to pT3 occurred in 61% of patients. Overall, 9.8% of cases exhibited positive surgical margins (PSMs), most of them single and limited in extent. Postoperative major complications were recorded in 6.3% of patients. At a median follow-up of 18 months (IQR: 6–29), biochemical recurrence (BCR) occurred in 8% of patients. Patients with PIRADS 5 lesions experienced shorter BCR-free survival compared to those with PIRADS 3–4. On multivariable Cox regression, PIRADS 5 independently predicted biochemical recurrence (HR: 2.52; 95% CI: 1.10–5.80; *p* = 0.029), after adjustment for age, number of lesions, and nerve-sparing status, with the performance of nerve-sparing not associated with an increased risk of recurrence, including in patients with multifocal disease. **Conclusions**: Nerve-Sparing Robot-Assisted Radical Prostatectomy in patients with multiple PIRADS lesions achieves encouraging short-term oncologic outcomes, with biochemical recurrence-free survival exceeding 84% at 3 years, despite high rates of multifocality and pathological upstaging.

## 1. Introduction

The optimal diagnostic and surgical management of patients with prostate cancer (PCa) still represents a major issue also due to its burden on global healthcare systems [1]. In this scenario, the introduction of Multiparametric Magnetic Resonance Imaging (mpMRI) has reshaped the diagnostic pathway, enabling the early detection and characterization of clinically significant tumors with high accuracy levels [2]. Nevertheless, although mpMRI has a well-established role in identifying index lesions, its reliability in the context of multifocal disease is still to be determined, thus hindering the preoperative risk stratification process and patient counselling [3,4,5]. In recent decades, the surgical management of PCa has undergone a paradigm shift, with the adoption of robot-assisted radical prostatectomy (RARP) becoming the standard of care in most tertiary referral centers [6,7]. This was mainly driven by several consistent advantages over the open approach in terms of perioperative safety, blood loss, and length of hospital stay while maintaining comparable oncological outcomes [8]. In this light, the adoption of a nerve-sparing (NS) technique might be enhanced due to several features inherent in the robotic platform, such as the magnified 3D vision, the wider range of motion and the more precise cautery provided by robotic instruments [9]. However, the feasibility and safety of NS-RARP remains highly debated in patients with multiple PIRADS lesions on mpMRI, thus potentially jeopardizing the functional outcomes [10]. Most large series and guidelines provide general recommendations based on risk group, PIRADS score, or clinical stage, but few studies have specifically addressed patients with multiple PIRADS lesions or confirmed multifocality at final pathology.

To fill this gap, we sought to evaluate a consecutive cohort of patients with multiple PIRADS lesions undergoing RARP in a high-volume tertiary referral center with a centralized imaging revision.

## 2. Materials and Methods

### 2.1. Study Design

After Institutional Review Broad approval, we prospectively collected the clinical and surgical data of consecutive patients treated with RARP for PCa between January 2020 and April 2023 at our tertiary referral center. The study was conducted in accordance with the ethical principles of the Declaration of Helsinki and all patients signed a written informed consent before enrolment.

The main inclusion criteria were the presence of an mpMRI performed and evaluated or re-evaluated by two expert uro-radiologists (S.A., F.D.N.) at our center, an MRI-ultrasound fusion biopsy performed by expert urologists or uro-radiologists at our center, and having completed a minimum follow-up. The main exclusion criteria were as follows: (1) Previous therapy for PCa, including RT, focal therapy or androgen deprivation therapy (ADT); (2) Evidence of distant metastasis, including non-regional lymph nodes, at preoperative conventional staging, consisting in bone scintigraphy (BS) and computed tomography (CT), or PET-PSMA. These exams were performed in all included patients; (3) mpMRI or ultrasound fusion biopsy performed in another center or performed at our center by other operators with limited expertise. No minimum follow-up duration was required for inclusion; however, patients without any postoperative follow-up data or with follow-up <12 months and no recorded outcome were excluded. Follow-up time was defined from surgery to last clinical assessment or biochemical recurrence. Time-to-event analyses were performed using Kaplan–Meier estimates, with patients censored at last follow-up if no event occurred. Included patients were stratified according to the preservation of bundle: radical (Group A), monolateral nerve-sparing (NS) (Group B), or bilateral NS (Group C). A subgroup analysis on upstaging and BCR outcomes based on surgical approach was performed.

### 2.2. Surgical Technique

All procedures were performed by four experienced surgeons (≥100 RARP), with a standard six ports transperitoneal configuration. The Da Vinci surgical system Si, X or Xi (Intuitive Surgical, Sunnyvale, CA, USA) was used throughout the cases. In cases of anticoagulant/antiplatelet (AC/AP) therapy, its suspension, replacement or maintenance was agreed with the anesthesiologist and the patient. An anterograde approach was chosen in all cases. Concomitant standard pelvic lymph node dissection (PLND) was performed according to the Briganti nomogram following standard anatomical landmarks, including the removal of lymphatic tissue along the external iliac vessels, within the obturator fossa, and around the internal iliac vessels. NS was performed with an intrafascial dissection between the peri-prostatic veins and the pseudocapsule of the prostate, or interfascial dissection along the peri-venous plane; or extrafascial dissection.

According to demographic, clinical and pathological features, NS side and technique were performed according to surgeon preference, also discussed and agreed with the patient prior to the surgery. Change in strategy of NS technique during treatment was also reported. Pelvic drainage was placed at the end of the procedure at surgeon’s discretion. A 18Ch Foley catheter was inserted during the procedure in all cases. Catheter removal was scheduled according to the surgeon’s judgment.

### 2.3. Covariates and Outcomes

The following clinical, imaging, and pathological variables were prospectively collected: patient age, body mass index (BMI), Charlson Comorbidity Index (CCI), presence of hypertension or diabetes mellitus, smoking status (categorized as current, former, or never smoker), history of previous transurethral resection of the prostate (TURP), and previous major abdominal surgery. Preoperative evaluation included serum prostate-specific antigen (PSA) levels and findings on digital rectal examination (DRE) performed by the attending urologist. Multiparametric MRI (mpMRI) parameters included prostate volume, Prostate Imaging Reporting and Data System (PI-RADS) score of the dominant lesion, maximum diameter of the index lesion, anatomical location (apex, mid-gland, base), and zonal distribution (anterior, peripheral, transitional zones). Radiological features suggestive of extracapsular extension (ECE) and seminal vesicle invasion (SVI) were systematically assessed, with ECE defined by the presence of neurovascular bundle asymmetry, capsular bulge or irregularity, measurable extracapsular disease, or obliteration of the rectoprostatic angle on high-resolution T2-weighted images, and SVI based on direct tumor extension into seminal vesicles, low signal intensity, or abnormal contrast enhancement. In the absence of these features, organ-confined disease was assumed. Biopsy variables included ISUP grade group assignment according to the modified Gleason scoring system endorsed by the 2005 and 2014 ISUP consensus conferences, and clinical staging (cT and cN) was determined based on DRE and imaging findings. PSA persistence was defined as a PSA ≥ 0.1 ng/mL within 4 to 6 weeks after surgery. Biochemical recurrence (BCR) was defined as a total PSA ≥ 0.2 ng/mL at two consecutive evaluations. UI was defined as the use of >1 pad per day. ED was defined as a IIEF-5 score < 12 (moderate or severe).

### 2.4. Follow-Up

First postoperative assessment included catheter removal; Kegel exercises were routinely illustrated concomitantly. Therefore, a postoperative assessment was scheduled approximately 30 days after surgery, and included clinical, histopathological and laboratory evaluation. Patients interested in erectile function recovery who underwent NS-RARP were provided with PDE5i treatment, which was offered free of charge by the National Health System [11]. The International Index of Erectile Function-5 (IIEF-5) was self-administered to assess sexual function. Therefore, patients were followed up at 3, 6 and 12 months postoperatively, and then twice a year. Total PSA, urinary incontinence (UI) and erectile disfunction (ED) were evaluated.

### 2.5. Statistical Analysis

Continuous variables are reported as median and interquartile range (IQR), while categorical variables are presented as frequencies and percentages. Between-group comparisons were performed using Student’s *t*-test or Mann–Whitney U test for continuous variables, according to data distribution assessed by the Kolmogorov–Smirnov test, and the chi-square test or Fisher’s exact test were used for categorical variables, as appropriate.

The primary oncological endpoint was biochemical recurrence (BCR), defined as a prostate-specific antigen (PSA) value ≥0.2 ng/mL confirmed by two consecutive measurements. Time-to-event analyses were performed using the Kaplan–Meier method to estimate BCR-free survival (BCR-FS), and differences between groups were assessed using the log-rank test. Patients were censored at the time of last follow-up if no event occurred.

To identify predictors of BCR, univariable and multivariable Cox proportional hazards regression models were used, reporting hazard ratios (HRs) with 95% confidence intervals (CIs). Variables included in the multivariable model were selected based on clinical relevance and data availability, and included age, preoperative PSA, biopsy ISUP grade group, clinical T stage, dominant lesion PI-RADS score, number of lesions, and nerve-sparing status. Given the limited number of events, model overfitting was minimized by restricting the number of covariates and prioritizing clinically meaningful variables.

The proportional hazards assumption was assessed using Schoenfeld residuals. Missing data were handled using complete-case analysis, given the low proportion of missingness. A two-sided *p*-value < 0.05 was considered statistically significant. All statistical analyses were performed using SPSS version 27 (IBM Corp., Armonk, NY, USA).

## 3. Results

Overall, 286 patients with multiple PIRADS lesions treated with RARP at a tertiary referral center were included (Supplementary Table S1). Nerve-sparing was not performed in 99 (34.6%) patients while unilateral and bilateral nerve-sparing were achieved in 63 (22.3%) and 124 (43.1%) patients, respectively. Median age was 69 years (IQR: 64–72), median BMI was 26.1 kg/m^2^ (IQR: 24.1–28) while median Charlson Comorbidity Index was 3 (IQR: 2–4). In terms of baseline tumor features, median preoperative total PSA was 6.7 ng/mL (IQR: 5.1–9.6), and a positive digital rectal examination (DRE) was present in 54.5% of cases (Table 1). No significant differences in baseline features were observed between nerve-sparing groups, although patients undergoing non-nerve-sparing surgery presented with a slightly higher prevalence of cT3 disease at staging. The presence of two PIRADS lesions was found in 81.8% of cases, while 18.2% presented with three or more (Table 2). Bilateral lesions were observed in 67.4% of cases. The dominant lesion was PIRADS 4 in 57.3% and PIRADS 5 in 32.3% of cases, with a median diameter of 12 mm (IQR: 10–17). Satellite lesions had a median diameter of 8 mm (IQR: 6–11). Concerning the pathological biopsy characteristics, an ISUP ≥ 3 lesion was observed in 33.7% of cases, while at final pathology ISUPs ≥ 3 and ≥4 were recorded in 31% and 25%, respectively, with a multifocal disease confirmed in the 46.3% of cases (Table 3). A subgroup analysis on upstaging and BCR outcomes based on surgical approach was performed. Pathological upstaging to pT3 occurred in 61% of patients. Overall, 9.8% of cases exhibited positive surgical margins (PSMs), most of them single and <5 mm. The dominant PIRADS lesion corresponded to the principal tumor site at histology in 82.1% of patients, while concordance between PIRADS score and highest ISUP grade was 72.5% (Table 4). Lymph node dissection was performed in 50.7%, with positive nodal disease detected in 6.6%. Intraoperative complications occurred in 1.4% of cases, while postoperative major complications were recorded in 6.3% of patients. Median length of stay was 3 days (IQR: 3–4). At a median follow-up of 18 months (IQR: 6–29), biochemical recurrence (BCR) occurred in 8% of patients. Patients with PIRADS 5 lesions experienced shorter BCR-free survival compared to those with PIRADS 3–4. On multivariable Cox regression, PIRADS 5 independently predicted BCR (HR: 2.52; 95% CI: 1.10–5.80; *p* = 0.029), after adjustment for age, number of lesions, and nerve-sparing status (Table 5). Importantly, the performance of nerve-sparing, whether unilateral or bilateral, was not associated with an increased risk of recurrence, including in patients with multifocal disease.

## 4. Discussion

The present study provides a comprehensive analysis of men with multiple PIRADS lesions undergoing RARP, highlighting both the diagnostic value of mpMRI and the oncological safety of nerve-sparing in multifocal disease.

The first key observation is the concordance between PIRADS findings and final pathology. In our series, the dominant PIRADS lesion corresponded to the index tumor at histology in 82.1% of patients. In this context, previous series without an imaging-central revision reported concordance rates ranging between 55% and 70%, with lower rates reported in case of multifocal disease [12,13]. Moreover, in our series the PIRADS score maintained a strong predictive value for tumor aggressiveness, as concordance between PIRADS score and ISUP grade was more than the 70%. This underlines the role of mpMRI not only as a localization tool but also as a prognostic marker of biological aggressiveness. From a clinical perspective, these results highlight the key role of an expert uro-radiologist’s imaging evaluation in everyday clinical practice, thus showing how the enhanced predictive role of the PIRADS score in high-volume centers is strictly related to a more precise patient selection process.

A second important finding relates to oncological outcomes. Despite the high prevalence of adverse pathological features in our cohort—including multifocality in nearly half of patients and pathological upstaging to pT3 in more than 60%—the biochemical recurrence-free survival remained favorable, exceeding 84% at 1 years. This underscores the safety and feasibility of RARP as an oncological treatment even in complex cases. Crucially, patients with multiple PIRADS lesions did not demonstrate significantly worse BCR-FS as compared to those with single lesions [14,15]. Moreover, while PIRADS 5 was an independent predictor of recurrence, the overall recurrence rate remained low, suggesting that the burden of lesions per se does not necessarily compromise short-term oncological control when surgery is performed by experienced hands [16,17,18].

The third key finding was that the adoption of a nerve-sparing approach was feasible and safe in patients with multifocal disease, thus optimizing functional outcomes without compromising oncological safety. Indeed, two-thirds of patients underwent either unilateral or bilateral nerve-sparing without the evidence of increased risk of recurrence after COX regression. This is of particular significance given persistent concerns in the adoption of a nerve-sparing strategy in patients with multiple lesions or high PIRADS scores [19,20]. In this scenario, our data indicate that, with appropriate patient selection and surgical expertise, nerve-sparing remains feasible and oncologically safe, even in cases of multifocal disease. From a translational perspective, these findings support the evolving role of PIRADS not only as a diagnostic but also as a prognostic instrument. While PIRADS cannot perfectly localize the index lesion in multifocal disease, higher scores remain strongly predictive of recurrence risk. This prognostic role should be incorporated into clinical discussions and may help refine follow-up protocols and adjuvant treatment strategies. Moreover, The high rate of pathological upstaging observed in our cohort should be interpreted in light of both patient selection and the intrinsic limitations of mpMRI. Indeed, this was not a low-risk population, as nearly half of patients were classified as EAU high-risk and more than half had a positive DRE at baseline. Moreover, final pathology confirmed a substantial burden of locally advanced disease, with pT3a and pT3b detected in 53.5% and 14.1% of cases, respectively. These findings suggest that centralized mpMRI evaluation may support surgical planning, but cannot fully exclude microscopic extracapsular extension in multifocal disease.

The present manuscript is not devoid of limitations. Firstly, the retrospective design and the single center setting might lead to intrinsic selection biases. Nevertheless, to the best of our knowledge the present manuscript represents the largest series of patients with multifocal diseases and a central imaging revision reported in the literature. Moreover, the lack of functional outcomes might reduce the reliability of reported results. In this light our aim was to assess the surgical safety of performing an NS approach in the multifocal disease patient setting. The relatively short oncological follow-up allowed us to evaluate only “early” results while long-term oncological durability remains to be proven. Given the relatively short median follow-up and the limited number of recurrence events, the estimated 2- and 3-year BCR-free survival rates should be interpreted with caution. In particular, the number of patients at risk decreases over time, potentially affecting the robustness of longer-term estimates. Therefore, our findings should be considered representative of early oncological outcomes only, and longer follow-up is required to confirm durability. The lack of a preoperative standardized definition of NS along with the absence of a codified distance between the index lesion and the neurovascular bundle might have introduced a selection bias. Lesion-specific pathologic–radiologic margin mapping was also not collected through our dataset and should be a focus of future studies. Finally, our findings are derived from a single high-volume center with standardized imaging and surgical expertise, and results may not be directly generalizable to centers with lower caseloads or less specialized imaging interpretation.

## 5. Conclusion

In conclusion, this study demonstrates that RARP in patients with multiple PIRADS lesions achieves encouraging short-term oncologic outcomes, with biochemical recurrence-free survival exceeding 84% at 3 years, despite high rates of multifocality and pathological upstaging. These findings support a more confident application of nerve-preserving strategies in multifocal prostate cancer.

## Figures and Tables

**Table 1:** Baseline clinical and oncological features of patients with positive multiparametric MRI and multiple PIRADS lesions ≥ 3 treated with Robot-Assisted Radical Prostatectomy at a tertiary referral center.

		Patients with Multiple PIRADS Lesions Treated with RARP N = 286
Family history PCa, *n (%)*		56 (19.9)
Age, *median (IQR)*		69 (64–72)
BMI, *median (IQR)*		26.1 (24.1–28)
Hypertension, *n (%)*		195 (68.2)
Diabetes mellitus, *n (%)*		139 (48.6)
Previous Abdominal surgery, *n (%)*		101 (35.4)
Previous prostate surgery, *n (%)*		25 (8.6)
Charlson Score comorbidity index, *n %*	0	6 (2.1)
	1	24 (8.4)
	2	88 (30.8)
	3	90 (31.5)
	4	54 (18.5)
	5	23 (8)
	6	2 (0.7)
Preoperative total PSA, *median (IQR)*		6.7 (5.1–9.6)
Prostate Volume (cc), *median (IQR)*		48 (36.2–62)
Positive DRE, *n (%)*		155 (54.5)
Clinical T, *n (%)*	cT1b	1 (0.4)
	cT1c	123 (43.8)
	cT2a	6 (2.1)
	cT2b	43 (15.3)
	cT2c	94 (33.5)
	cT3a	4 (1.4)
	cT3b	4 (1.4)
Eau risk group, *n (%)*	Low risk	4 (1.4)
	Intermediate	146 (51.8)
	High risk	132 (46.8)

**Table 2:** Preoperative and multiparametric MRI features of patients with multiple PIRADS lesions > 3 treated with Robot-Assisted Radical Prostatectomy at a tertiary referral center.

		Patients with Multiple PIRADS Lesions Treated with RARP N = 286
PIRADS location, *n (%)*	Monolateral Right	30 (10.9)
	Monolateral Left	60 (21.7)
	Bilateral	186 (67.4)
Number of PIRADS lesion, *n (%)*	2	234 (81.8)
	3	44 (15.4)
	4	5 (1.7)
	5	3 (1)
Highest PIRADS lesion, *n (%)*	3	29 (10.1)
	4	164 (57.3)
	5	92 (32.3)
Dimension of highest PIRADS lesion (mm), *median (IQR)*		12 (10–17)
Dimension of the second PIRADS lesion (mm), *median (IQR)*		8 (6–11)
Fusion Biopsy technique, *n (%)*	Transperineal Biopsy	157 (54.9)
	Transrectal Biopsy	129 (45.1)
Biopsy ISUP grade, *n (%)*	1	53 (18.8)
	2	133 (47.2)
	3	48 (17)
	4	34 (12.1)
	5	14 (5)
Number of cores, median (IQR)		12 (8–14)
Positive core, median (IQR)		4 (3–6)
Total cancer core length (mm), median (IQR)		20 (9–37)

**Table 3:** Perioperative features of patients with positive multiparametric MRI and multiple PIRADS lesions ≥ 3 treated with Robot-Assisted Radical Prostatectomy at a tertiary referral center.

			Patients with Multiple PIRADS Lesions Treated with RARP N = 286
Nerve-sparing surgery, *n (%)*	Radical		99 (35.1)
	Monolateral		63 (22.3)
	Bilateral		120 (42.6)
Pelvic Lymph Node Dissection (PLND), *n (%)*			145 (50.7)
Console time (min), *median (IQR)*			113 (96–168)
Estimated Blood Loss (cc), *median (IQR)*			130 (60–250)
Intraoperative complication, *n (%)*			4 (1.4)
Postoperative Major complication (Clavien Dindo ≥ 3), *n (%)*	No		268 (93.7)
	Yes		18 (6.3)
LOS, *median (IQR)*			3 (3–4)
Blood transfusions, *n (%)*			6 (2.1)
90-day readmissions, *n (%)*			8 (2.8)
Pathology histology, *n (%)*		Acinar	241 (86.7)
		Ductal	22 (7.9)
		Cribriform	4 (1.4)
		Microcystic	9 (3.2)
		Neuroendocrine	2 (0.7)
Pathological T stage, *n (%)*		pT2	92 (32.4)
		pT3a	152 (53.5)
		pT3b	40 (14.1)
Pathological ISUP, *n (%)*		1	18 (6.6)
		2	137 (50)
		3	52 (19)
		4	35 (12.8)
		5	32 (11.79)
Pathological N stage, *n (%)*		pNx	137 (47.9)
		pN0	131 (45.8)
		pN1	18 (6.3)

**Table 4:** Oncological features of patients with positive multiparametric MRI and multiple PIRADS lesions ≥ 3 treated with Robot-Assisted Radical Prostatectomy at a tertiary referral center.

		Patients with Multiple PIRADS Lesions Treated with RARP N = 286
Positive Surgical Margin (PSMs), *n (%)*		28 (9.8)
Number of PSMs, *n (%)*	1	21 (7.3)
	2	5 (1.7)
	3	2 (0.6)
PSM length (mm), *median (IQR)*		3 (2–4)
Early Post operative total PSA persistence, *n (%)*		18 (6.3)
Biochemical Recurrence 6 months, *n (%)*		4 (1.4)
Biochemical Recurrence 12 months, *n (%)*		8 (2.8)
Biochemical Recurrence 36 months, *n (%)*		3 (1.0)
Follow-Up (month), *median (IQR)*		18 (6–29)

**Table 5:** MultivariaCox regression analysis assessing independent predictors of disease recurrence in patients with positive multiparametric MRI and multiple PIRADS lesions > 3 treated with Robot-Assisted Radical Prostatectomy at a tertiary referral center.

Covariates		HR	95% CI	*p* Value
Age (years)		1.003	0.87–1.04	0.07
Main lesion PIRADS score				
	3	Ref		
	4	1.34	0.72–2.45	0.2
	5	2.52	1.10–5.80	0.02
Number of lesions				
	2	Ref		
	3	1.2	0.85–2.73	0.4
	>3	1.02	0.76–5.81	0.2
PIRADS dimension (mm)		1.01	1.007–1.24	0.02
Nerve-Sparing Technique				
	Radical	Ref		
	Monolateral	1.12	0.84–1.68	0.19
	Bilateral	1.38	0.91–1.72	0.08

## Data Availability

The original contributions presented in this study are included in the article. Further inquiries can be directed to the corresponding author.

## Supplementary Materials

The following supporting information can be downloaded at: https://www.mdpi.com/article/10.3390/jcm15103787/s1, Table S1. Flow chart showing patients inclusion with positive multiparametric MRI and multiple PIRADS lesions ≥ 3 treated with Robot Assisted Radical prostatectomy at a tertiary referral center.
