# Supplementary material for: Evaluating Oncological Outcomes in Patients with Multiple PiRADS Lesion Treated with Robot-Assisted Radical Prostatectomy for Prostate Cancer: Results from a Large Contemporary Cohort with Centralized MpMRI Evaluation in a High-Volume Center
Source: J Clin Med. 2026 May 14;15(10):3787. doi: 10.3390/jcm15103787 (PMC13207318; doi:10.3390/jcm15103787)
Supplement: Supplementary file 1 [file jcm-15-03787-s001.zip › jcm-4248387-supplementary.pdf]

Patients undergoing RARP at our institution (Jan 2020 – Apr 2023)	n = [1876]
↓ Excluded:	- mpMRI not performed or not centrally reviewed<br>- Fusion biopsy not performed at our center<br>- Prior PCa treatment (RT, ADT, focal therapy)<br>- Metastatic disease at staging
↓ Eligible patients with centrally reviewed mpMRI and fusion biopsy	n = [989]
↓ Patients with multiple PI-RADS lesions on mpMRI	n = [286]
↓ Final study cohort included in analysis	n = [286]

Supplementary Table S1. Flow chart showing patients inclusion with positive multiparametric MRI and multiple PIRADS lesions $\geq$ 3 treated with Robot Assisted Radical prostatectomy at a tertiary referral center.
